# Reclassified the phenotypes of cancer types and construct a nomogram for predicting bone metastasis risk: A pan‐cancer analysis

**DOI:** 10.1002/cam4.7014

**Published:** 2024-03-01

**Authors:** Ming Li, Wenqian Yu, Chao Zhang, Huiyang Li, Xiuchuan Li, Fengju Song, Shiyi Li, Guoheng Jiang, Hongyu Li, Min Mao, Xin Wang

**Affiliations:** ^1^ Department of General Surgery, Section for HepatoPancreatoBiliary Surgery, The Third People's Hospital of Chengdu Affiliated Hospital of Southwest Jiaotong University & The Second Affiliated Hospital of Chengdu, Chongqing Medical University Chengdu China; ^2^ Department of Epidemiology and Health Statistics, West China Public Health School and West China Fourth Hospital Sichuan University Chengdu China; ^3^ Department of Bone and Soft Tissue Tumours National Clinical Research Center for Cancer, Key Laboratory of Cancer Prevention and Therapy, Tianjin's Clinical Research Center for Cancer, Tianjin Medical University Cancer Institute and Hospital Tianjin China; ^4^ Department of Cardiology General Hospital of Western Theater Command Chengdu P.R. China; ^5^ Department of Epidemiology and Biostatistics, Key Laboratory of Cancer Prevention and Therapy, Tianjin Key Laboratory of Breast Cancer Prevention and Therapy, Ministry of Education, National Clinical Research Center for Cancer Tianjin Medical University Cancer Institute and Hospital Tianjin People's Republic of China; ^6^ The Joint Laboratory for Lung Development and Related Diseases of West China Second University Hospital Sichuan University and School of Life Sciences of Fudan University, West China Institute of Women and Children's Health, West China Second University Hospital, Sichuan University Chengdu China

**Keywords:** associated factors, bone metastasis, cancer classification system, prediction nomogram

## Abstract

**Background:**

Numerous of models have been developed to predict the bone metastasis (BM) risk; however, due to the variety of cancer types, it is difficult for clinicians to use these models efficiently. We aimed to perform the pan‐cancer analysis to create the cancer classification system for BM, and construct the nomogram for predicting the BM risk.

**Methods:**

Cancer patients diagnosed between 2010 and 2018 in the Surveillance, Epidemiology, and End Results (SEER) database were included. Unsupervised hierarchical clustering analysis was performed to create the BM prevalence‐based cancer classification system (BM‐CCS). Multivariable logistic regression was applied to investigate the possible associated factors for BM and construct a nomogram for BM risk prediction. The patients diagnosed between 2017 and 2018 were selected for validating the performance of the BM‐CCS and the nomogram, respectively.

**Results:**

A total of 50 cancer types with 2,438,680 patients were included in the construction model. Unsupervised hierarchical clustering analysis classified the 50 cancer types into three main phenotypes, namely, categories A, B, and C. The pooled BM prevalence in category A (17.7%; 95% CI: 17.5%–17.8%) was significantly higher than that in category B (5.0%; 95% CI: 4.5%–5.6%), and category C (1.2%; 95% CI: 1.1%–1.4%) (*p* < 0.001). Advanced age, male gender, race, poorly differentiated grade, higher T, N stage, and brain, lung, liver metastasis were significantly associated with BM risk, but the results were not consistent across all cancers. Based on these factors and BM‐CCS, we constructed a nomogram for predicting the BM risk. The nomogram showed good calibration and discrimination ability (AUC in validation cohort = 88%,95% CI: 87.4%–88.5%; AUC in construction cohort = 86.9%,95% CI: 86.8%–87.1%). The decision curve analysis also demonstrated the clinical usefulness.

**Conclusion:**

The classification system and prediction nomogram may guide the cancer management and individualized BM screening, thus allocating the medical resources to cancer patients. Moreover, it may also have important implications for studying the etiology of BM.

## 1 INTRODUCTION

Bone metastasis (BM) is one of the most common sites for metastasis and is a leading cause of death in advanced cancer patients.^1^, ^2^ Furthermore, BM may lead to a range of known as skeletal‐related events (SREs), which include bone pain, pathologic fractures, hypercalcemia, spinal cord compression, and the need for palliative treatment for the bone.^3^ The unfavorable prognosis and SREs significantly affect the quality of life and inflicts heavy disease and economic burden to the cancer patients.^4^, ^5^ Despite the treatment options in oncology having a marked development during the past decades, there is still a lack of curable and standard treatment protocols for BM patients.^6^, ^7^, ^8^, ^9^ Hence, early prediction of the BM risk and promptly offering individualized screening and prophylactic treatment to high‐risk patients is critical in clinical BM management.^10^, ^11^, ^12^

A package of imaging examinations, including x‐rays, computed to‐myography (CT), magnetic resonance imaging, and positron emission tomography‐computed tomography has been developed to timely detect the BM occurrence, but the radiation exposure and the financial burden limit its extensive examination.^13^ Accordingly, efficient methods were warranted to accurately predict the BM risk and systematically manage the cancer patients. Although the anatomical system may be a potential choice due to the similar symptoms and pathogenic mechanism, pieces of studies in vain to verify the similar BM patterns even in different histological types of same cancer.^11^, ^14^ Genetics may also have deep value in forecasting BM risk, while the invasive inspection method, high‐cost and precision equipment‐dependent characteristics limit its wide application in the clinical practice.^15^, ^16^, ^17^

The identification of associated factors for BM will play an important role in the prediction of BM risk. Numerous articles have identified multiple risk factors for BM, leading to the development of several prediction models. Dong et al. established a predictive model to evaluate the risk of BM in kidney cancer and found that the comprehensive predictive tool, consisting of a nomogram and web calculator, contributes to risk stratification. This model helped clinicians identify high‐risk cases.^11^ Moreover, the research findings by Zhang et al. on the prediction model for BM in pancreatic cancer demonstrate that the column chart predictive model, incorporating variables such as age, N stage, and brain metastasis, exhibits excellent predictive performance (with an AUC of 85% in the external validation cohort).^18^ Additionally, machine learning techniques were also used in the model establishment and showed excellent performance with an AUC reaching 96.2%.^19^, ^20^ However, due to limitations in sample size, scope of the study, and the opacity of machine learning technology, the results were not consistent across studies.^11^, ^18^, ^19^, ^20^, ^21^ Furthermore, because of the complex variety of cancer types, it is difficult for clinicians and policymakers to use these models and allocate health care resources wisely and efficiently. Consequently, it is crucial to conduct a universally applicable BM risk prediction model for pan‐cancer types and take individualized and appropriate intervention measures in time to prevent or delay the occurrence of BM.

National Cancer Institute's Surveillance, Epidemiology, and End Results (SEER) program is an important data source for cancer epidemiological analyses, which was established in 1973, covering more than 5 million US cancer patients across various geographic regions. The present study aims to first conduct a pan‐cancer analysis of the epidemiological characteristics of BM and establish a BM prevalence‐based cancer classification system (BM‐CCS) using the SEER database. And then, a nomogram based on the BM‐associated factors and the BM‐CCS was constructed for predicting the individualized BM risk. Besides, we also develop open‐source software, available through a website, to facilitate clinicians and patients.

## 2 MATERIALS AND METHODS

### 2.1 Study population

The study population was recruited from the SEER database, which covers about 30% of the American population.^22^ The cancer patients diagnosed between 2010 and 2016 were recruited as the construction dataset, as the status of BM was not initially collected until 2010. The patients recruited between 2017 and 2018 in the SEER were regarded as the validation dataset. The flowchart of the population selection was listed in Appendix S1.

### 2.2 Ethics statement

The SEER is an open‐access database, the release of data from the SEER database does not require informed patient consent as cancer is a reportable disease in every state of the United States.

### 2.3 Statistical analysis

Numerical data such as age were summarized as median ± interquartile range. Categorical variables were presented as counts and percentages and the differences were tested by Pearson chi‐square test or rank‐sum test. The prevalence of BM for each cancer type was calculated as the percentage of the subjects with BM within the total number of cancer patients. For the heterogeneous BM prevalence across all types of cancer, the pooled BM prevalence was calculated by combining the prevalence of BM for different cancers using meta‐analysis.

Unsupervised hierarchical clustering analysis with the squared Euclidean distance method was performed based on the BM prevalence and classified these cancer types into A, B, and C categories. Subgroup analysis was conducted to analyze the differences in the pooled BM prevalence between different categories. The univariable logistic regression model was conducted to determine the associated factors for BM risk and the factors with *p* < 0.05 were incorporated into the multivariable regression model. Based on the identified associated factors, a BM‐predicting nomogram was constructed to predict the individualized BM occurrence risk. The calibration curve and receiver operating characteristics curve (ROC) were used to evaluate the performance of the predicting nomogram. Calibration ability was evaluated by plotting the nomogram‐predicted BM probability versus the actual BM probability for patients by bootstrapping with 1000 resamples and the Hosmer and Lemeshow test. It can be considered that the predictive model has good calibration when the *p*‐value >0.05 for the Hosmer‐Lemeshow test. The discrimination of the nomogram was evaluated by the receiver operating characteristics curve (ROC). The area under the ROC of 0.5 indicated no discrimination and a value of 1.0 indicated the perfect separation of patients. Decision curve analysis (DCA) was also used to evaluate the clinical benefits and utility of the BM‐predicting nomogram by calculating the net benefits under differential threshold probabilities.^23^ External validation was conducted to examine the generalizability of the cancer classification system and the predicting nomogram in the SEER dataset diagnosed between 2017 and 2018.

SEER*Stat Software version 8.3.9.2 (https://seer.cancer.gov/seerstat/) (Information Management Service, Inc., Calverton, MD, USA) was used to generate the case list. Statistical analyses were carried out using Statistical Package for the Social Sciences (SPSS) version 23.0 software package for Windows (SPSS Inc) and R version 4.1.2 (R Foundation for Statistical Computing, Vienna, Austria; www.r‐project.org). Statistically significant levels were two‐tailed and set at *p* < 0.05.

## 3 RESULTS

### 3.1 Characteristics of the included cancer patients

A total of 50 cancer types incorporating 2,438,680 patients were included in the construction dataset, median age of the participants was 65.0 ± 18.0 years, 49.4% were males (*N* = 1,203,836) and 80.5% were white race (*N* = 1,962,889). The demographic and clinical characteristics of these patients were shown in Appendix S2.

For the validation dataset, a total of 281,041 records fulfilled the inclusion criteria. The median age of the participants was 66.0 ± 17.0 years, 49.6% were males (*N* = 139,467) and 78.0% were white race (*N* = 219,210). The distribution of demographic and clinical characteristics for the construction and validation dataset were shown in Appendix S3.

### 3.2 Prevalence of BM for all cancer types

**FIGURE 1:**
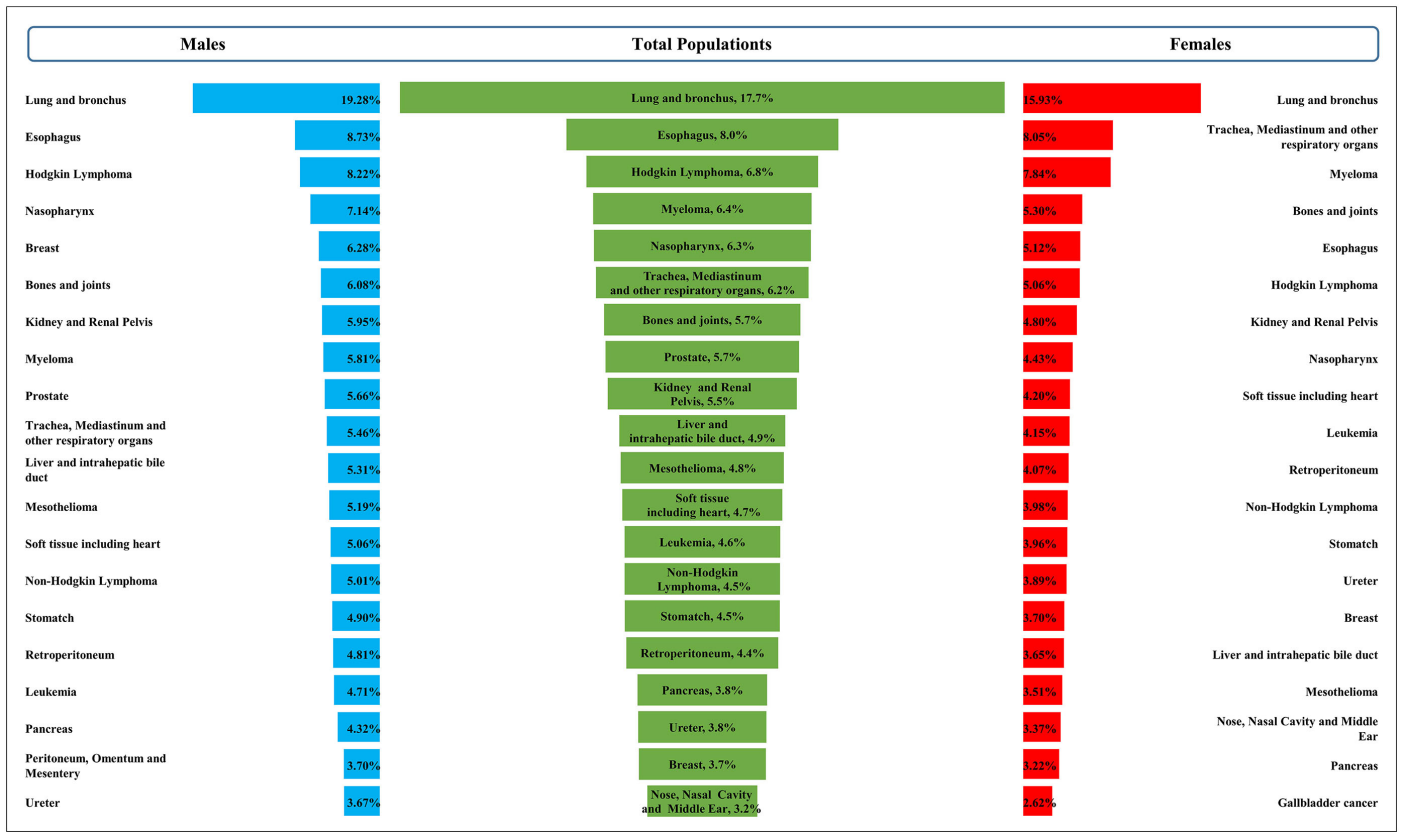
Spectrum distribution for top 20 bone metastasis prevalence cancer types among total, male and female patients.

A total of 124,316 cancer patients were diagnosed as BM at admission and different cancer types showed inconsistent BM prevalence. The prevalence of BM in the total population was highest in Lung and bronchus cancer (17.7%; 95% CI: 17.5%–17.8%), followed by Esophagus (8.0%; 95% CI: 7.6%–8.3%) and Hodgkin lymphoma (6.8%; 95% CI: 5.8%–8.0%), while the Brain cancer demonstrated the lowest BM prevalence (0.2%; 95% CI: 0.1%–0.3%). When stratified by sex, lung and bronchus cancer was listed as the top one BM prevalence for males and females. However, the spectrum distribution for the other 19 cancers with top BM prevalence was inconsistent between males and females. (Figure 1).

**FIGURE 2:**
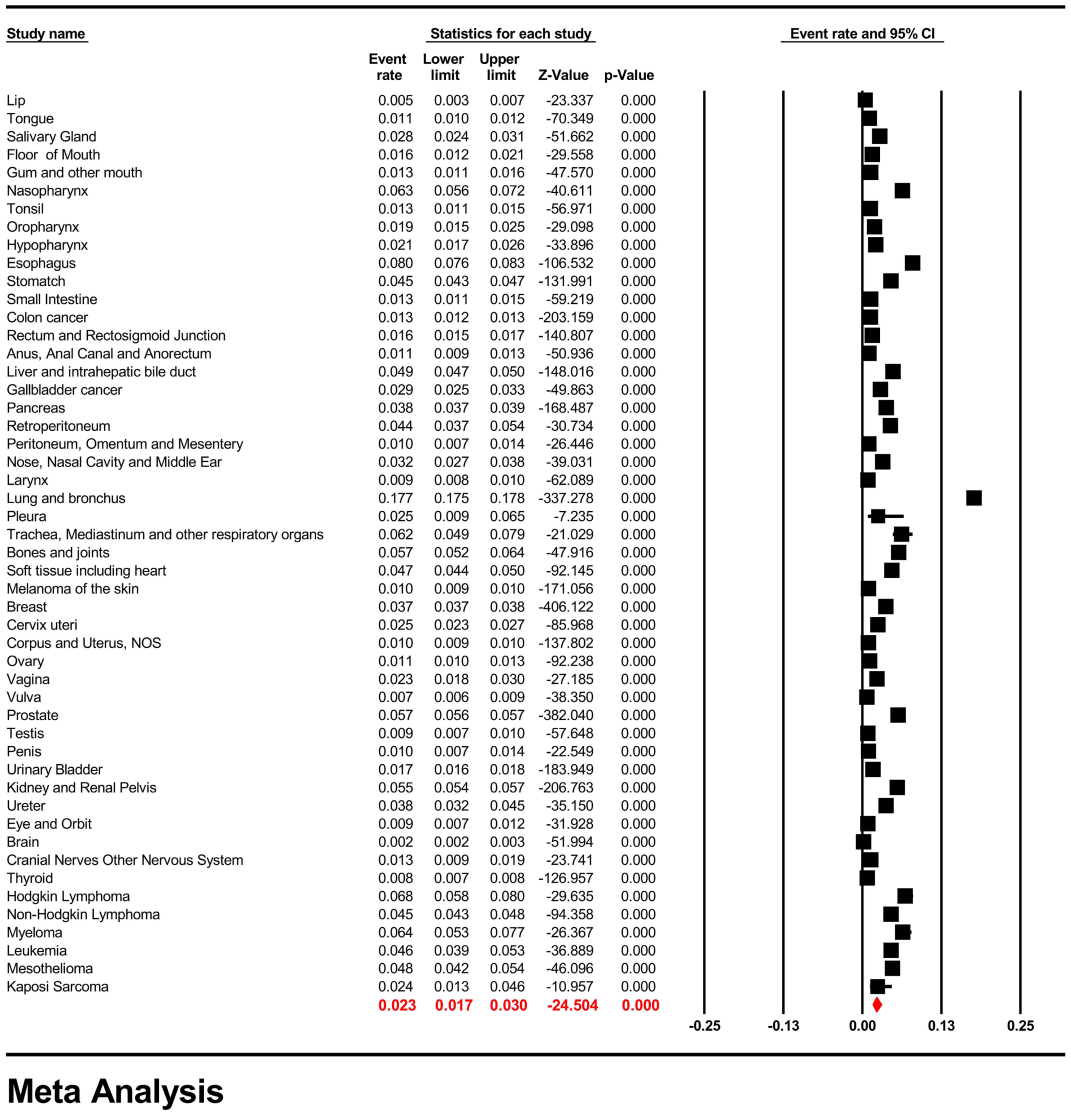
Forest plot for the pooled prevalence of bone metastasis across all of the cancer types.

Meta‐analysis suggested the pooled BM prevalence was 2.3% (95% CI: 1.7%–3.0%) and that in male and females were 2.7% (95% CI: 2.9%–3.6%) and 2.3% (95% CI: 1.7%–3.2%), respectively with no significant difference (*p* = 0.21). (Figure 2, Appendix S4) Meta‐regression suggested the pooled BM prevalence was significantly increased with year (*p* for slope = 0.019). (Appendix S5).

### 3.3 BM prevalence‐based cancer classification system (BM‐CCS)

**FIGURE 3:**
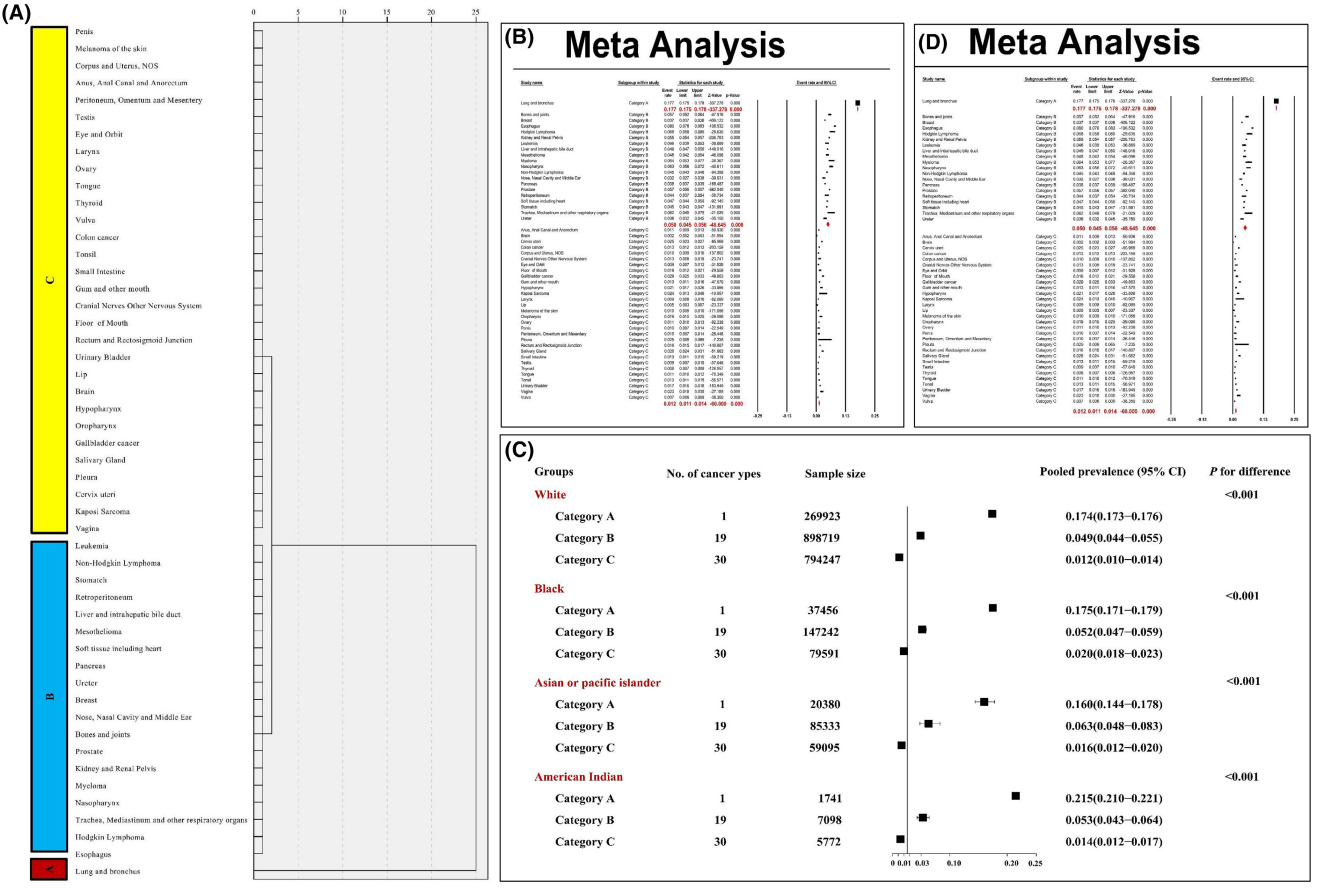
Unsupervised hierarchical cluster analysis for the classification of cancer types into three categories based on bone metastasis prevalence (A); the differences in the pooled bone metastatic prevalence among these three categories in the construction cohort (B), and stratified by different races (C), and in the validation cohort (D).

Unsupervised hierarchical clustering analysis classified the 50 cancer types into three main phenotypes, namely, category A, B, and C. (Figure 3A) Category A included the lung and bronchus cancer which indicated the highest BM prevalence (pooled BM prevalence = 17.7%; 95% CI: 17.5%–17.8%). Category B with moderate BM prevalence (pooled BM prevalence = 5.0%; 95% CI: 4.5%–5.6%) included bones and joints, breast, esophagus, Hodgkin Lymphoma, kidney and renal pelvis, leukemia, liver and intrahepatic bile duct, mesothelioma, myeloma, nasopharynx, non‐Hodgkin lymphoma, nose, nasal cavity and middle ear, pancreas, prostate, retroperitoneum, soft tissue including heart, stomach, trachea, mediastinum and other respiratory organs, and ureter cancer. Category C covers anus, anal canal, and anorectum, brain, cervix uteri, colon cancer, corpus and uterus, cranial nerves and other nervous system, eye and orbit, floor of mouth, gallbladder cancer, gum and other mouths, hypopharynx, Kaposi sarcoma, larynx, lip, melanoma of the skin, oropharynx, ovary, penis, peritoneum, omentum and mesentery, pleura, rectum, and rectosigmoid junction, salivary gland, small intestine, testis, thyroid, tongue, tonsil, urinary bladder, vagina, vulva showed lowest BM prevalence (pooled BM prevalence = 1.2%; 95% CI: 1.1%–1.4%) (*p* for difference <0.001). (Figure 3B) Significant differences in the pooled BM prevalence were also founded between different categories when stratified by the races of the included participants. (Figure 3C) Moreover, the differences in the pooled BM prevalence among these three categories were also confirmed in the validation dataset (*p* for difference <0.001). (Figure 3D).

Significant differences were found in the demographic and clinical characteristics among different categories and category A presented significantly higher percentage of older age (*χ* ^2^ = 11479.41; *p* < 0.001), unmarried (*χ* ^2^ = 11118.15; *p* < 0.001) brain metastasis (*χ* ^2^ = 133620.64; *p* < 0.001), liver metastasis (*χ* ^2^ = 13436.48; *p* < 0.001), lung metastasis (*χ* ^2^ = 50420.14; *p* < 0.001) and poor differentiated grade (*Z* = 7726.01; *p* < 0.001), higher T (*Z* = 74638.26; *p* < 0.001) and N stage (*Z* = 181162.98; *p* < 0.001) than the other categories, while category C showed highest proportion of female gender (*χ* ^2^ = 12267.17; *p* < 0.001), white race (*χ* ^2^ = 6918.44; *p* < 0.001) and uninsured status (*χ* ^2^ = 5339.05; *p* < 0.001). (Appendix S6).

### 3.4 Associated factors for developing BM

**FIGURE 4:**
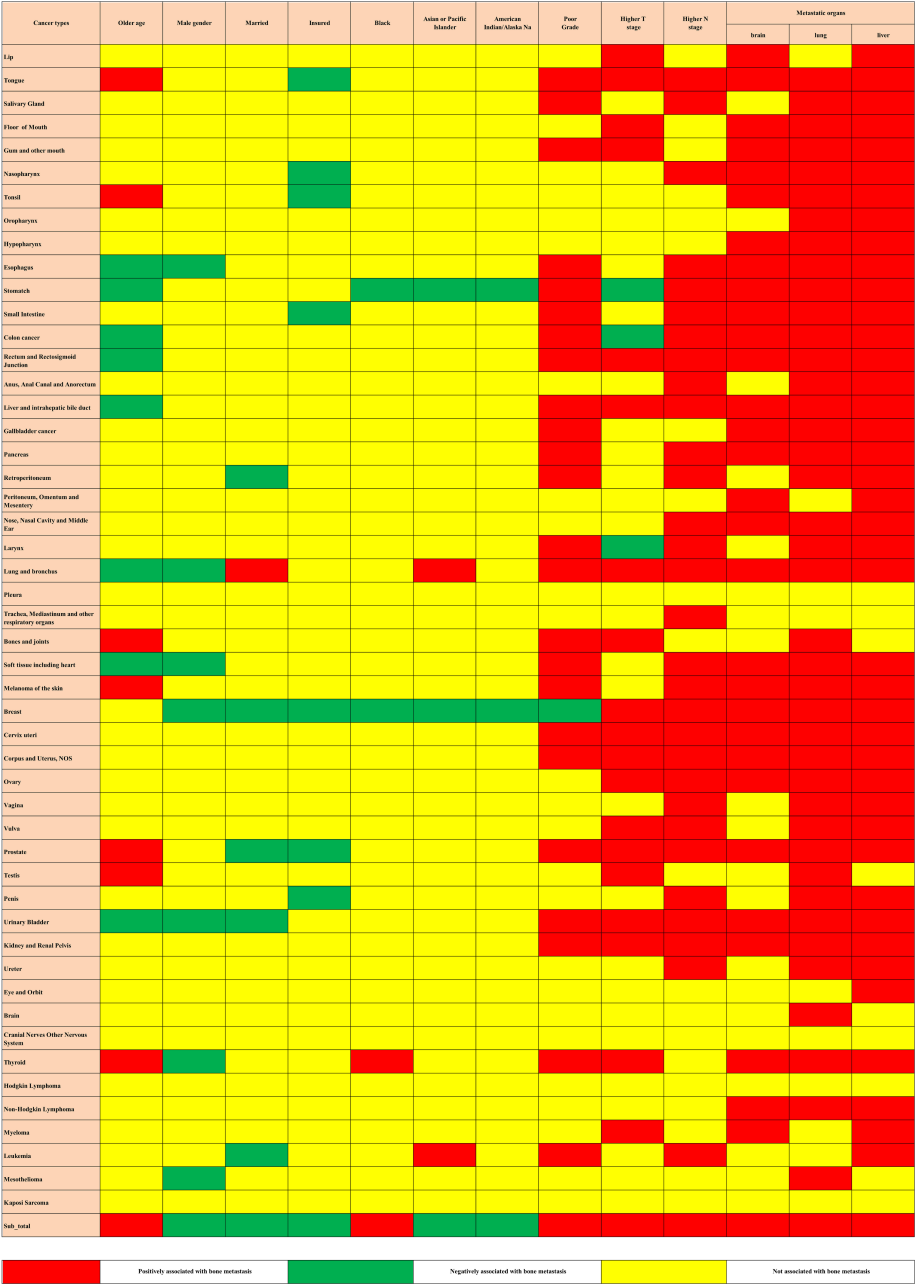
Risk factors for bone metastasis in the construction cohort. The red color and green color describe risk factors and protective factors for the bine metastatic risk, respectively, while the yellow color indicates that the factors did not reach the significance level.

Multivariable logistic regression showed advanced age, male gender, Black race, poorly differentiated grade, higher T stage, higher N stage, and brain, lung, and liver metastases were all positively associated with BM risk, while female gender, married status, insured status, Asian or Pacific Islander and American Indian race were all negatively related to BM risk and these associations were not consistent across all of the cancer types (Figure 4).

When further incorporated the prevalence‐based cancer classification system into the multivariable logistic regression model, the associations between these demographic and clinical characteristic factors and BM risk were not significantly altered, moreover, results showed category B [odds ratio (OR) = 0.68; 95% CI: 0.66–0.70; *p* < 0.001] and category C (OR = 0.14; 95% CI: 0.13–0.15; *p* < 0.001) were negatively correlated with the BM risk when compared with category A. (Appendix S7).

### 3.5 Construction and validation of the BM‐predicting nomogram

**FIGURE 5:**
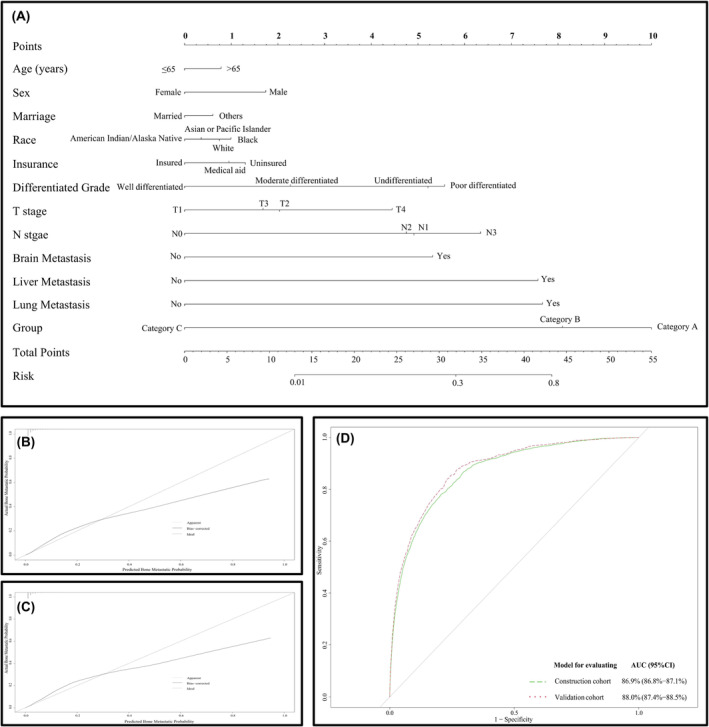
The nomogram for predicting the bone metastasis risk in the construction cohort (A); the calibration curve for validating the diagnostic accuracy of the nomogram in the construction cohort (B) and validation cohort (C) and the ROC curve for validating the discrimination ability of the nomogram in the construction and validation cohort (D).

A pan‐cancer‐based BM‐predicting nomogram integrated all of the significant factors was constructed for predicting the individualized BM risk (Figure 5A). The calibration curve revealed good agreement between the nomogram predicted and observed probabilities for BM occurrence, but there will be overestimation at a certain threshold (*p*‐value of H‐L test both in training and validation group <0.001) (Figure 5B). External validation also suggested prediction curve (solid line) of the calibration curve was closely approximated at the 45° line within a certain range of risk probabilities (Figure 5C). In addition, the nomogram exhibited good discrimination between patients with and without BM, and the area under the ROC curve was 86.9% (95% CI: 86.8%–87.1%) and 88.0% (95% CI: 87.4%–88.5%) in the construction and validation dataset, respectively (Figure 5D). Finally, the DCA was used to evaluate whether interventions based on our established predictive model would benefit patients with various cancers. It compared the net benefit of interventions based on model predictions with the net benefits of interventions for all or none of the patients. The results indicated that, within the threshold range of 0–0.9, the net benefit of clinical predictions and subsequent interventions using the model was greater than that of either intervening for all patients or not intervening at all. (Appendix S8) To facilitate the cancer patients and clinicians to predict the BM risk and guide the BM screening in the clinical practice, we established an open‐source software, through a website, (https://wangxinraine.shinyapps.io/Bone_Metastasis_Prediction/).

## 4 DISCUSSION

To the best of our knowledge, this study represents the first attempt to perform a pan‐cancer analysis involving approximately 2.5 million cancer patients. The aim was to delineate the epidemiological characteristics of BM, reclassify the phenotypes of various cancer types, and formulate the BM‐CCS. Furthermore, a predictive nomogram was developed using BM‐associated environmental factors and the BM‐CCS to forecast individualized BM risk.

Bone is one of the most common and lethal sites for metastatic growth across cancer types. For the included 50 cancer types, the BM prevalence was reported to be range from 0.2% to 17.7%, and different cancer types showed heterogeneous BM prevalence, even in the same anatomical system. The results may be partly explained by the “seed and soil” hypothesis.^24^, ^25^ The tumor cells were acted as “seeds” and the targeted organ has a friendly microenvironment as the “soil”, thus multiple types of cancer may harbor a specific ability to home to the bone microenvironment.^3^ Pieces of studies suggested that breast and prostate cancers were the most common malignancies that metastasize to bone in males and females, respectively.^1^, ^26^, ^27^ However, the current study demonstrated that, regardless of gender, the highest prevalence of BM was observed in lung and bronchus cancer. This discrepancy may stem from variations in the definitions of BM. In this study, we examined the prevalence of BM at admission (synchronous metastasis), whereas other studies focused on the occurrence of BM over a study period, referred to as “metachronous metastasis.”^1^, ^28^ These two conditions may reflect different features of the BM occurrence; however, seldom study tried to reveal the potential mechanism under the differences between them.

Additionally, to systematically draw the outline of BM prevalence and facilitate the cancer management for the clinicians, we established a cancer classification system (BM‐CCS) and redefined all of the cancer types into three main phenotypes based on the synchronous BM prevalence. Category A hold the highest BM prevalence while category C showed the lowest prevalence, and the differences among these phenotypes were not altered by race and the study population. The BM‐CCS, guided by the prevalence of BM, surpasses the limitations imposed by the anatomical system. It offers a convenient approach for clinicians and policymakers to oversee all cancer patients at high risk of BM and effectively allocate limited healthcare resources.

Moreover, we also found significant differences in the demographic and clinical characteristics among these three phenotypes, and category A prone to present a more advanced clinical stage and a higher proportion of organ metastasis than the other two phenotypes. Hence, we hypothesize that the high proportion of synchronous BM may be partly derived from the rapid cancer progression caused by the relatively higher malignancy degree and the inadequate and overdue BM screening.^11^, ^14^, ^21^ To provide timely and individualized BM screening, we explore the associated factors for BM occurrence and constructed a predicting nomogram.

Results showed different cancer types present homogenous and heterogeneous associated factors for BM development, the phenomenon may be explained by the inter‐and intra‐tumor heterogeneity that originated from genetic and non‐genetic factors.^29^ The multivariable logistic regression model suggested, the BM‐CCS was positively associated with BM risk, which was independent of the demographic and clinical risk factors for BM. Accordingly, we incorporated the BM‐CCS into the model and developed the first pan‐cancer risk prediction nomogram for synchronous BM at diagnosis. The internal validation showed the nomogram has good calibration and discrimination ability and the external validation also confirm its external applicability.

DCA puts together the benefit and harm to measure the net benefit of the BM‐predicting nomogram and proved it can serve as an excellent diagnostic tool for predicting BM. Compared with the ROC curve, the DCA takes clinical usefulness into the consideration, which is an important judging indicator of whether a prediction model can be truly used in clinical practice.^23^ In addition, to facilitate the clinical use of the BM‐predicting nomogram, we developed a website for the patients and doctors to evaluate the BM probability and conducted the BM screening timely.

There are now many predictive models that adopt machine learning methods, which exhibit better predictive performance. However, we still chose a nomogram‐based predictive model for the following reasons: First, our input variables do not include non‐linear relationships and complex high‐dimensional data. Additionally, since the primary purpose of establishing this predictive model is to rapidly screen individuals at high risk of BM across various cancer types, the weight of input variables in influencing the outcome is a crucial consideration in practice. Variables assigned higher scores in the nomogram are more deserving of attention in real‐life scenarios.^11^, ^21^, ^30^ If a variable is controllable, it can be beneficial for implementing targeted interventions for patients with various cancers, thereby reducing the risk of BM. Indeed, machine learning models exhibit predictive performance and accuracy that are superior to nomogram to some extent.^19^ However, due to their relatively lower interpretability, their application in clinical and public health domains will be subject to certain limitations.

Despite these advantages, there were several limitations in our study. First, the SEER only records part of the demographic and clinical characteristics, we could not thoroughly investigate all of the associated factors for BM, which may partly affect the performance of the predicting nomogram. Second, the SEER did not distinguish the specific bone metastatic site, we thus could not further predict the risk of BM at specific sites. Third, the construction and validation dataset set were all originated from the SEER database, the preliminary findings and predictive models should be further externally validated in other populations.

In conclusion, we conducted a pan‐cancer analysis of the prevalence and associated factors for BM and established a BM‐CCS to help redefined all of the cancer types into three phenotypes. Finally, we constructed a nomogram based on the BM‐CCS and other associated factors for predicting BM probability and validated the performance and clinical usefulness of the nomogram. This instrument could guide the individualized BM screening and help the clinicians and policymakers to develop BM screening strategies and policies to allocate health resources and prevent the patients from BM occurrence. Additionally, due to the intuitiveness and interpretability of the nomogram scoring process, the model we established was also advantageous in identifying modifiable variables related to BM in patients with various cancers. This facilitated the implementation of targeted personalized preventive measures. We believe that the application of this predictive model in clinical settings will contribute to the establishment of a disease stratification management system. Based on the model's predictive results, clinicians can conduct appropriate imaging or blood tests for high‐risk individuals, reduce screening frequency for moderate‐risk individuals, and implement routine monitoring measures for low‐risk individuals. In the end, we also develop open‐source software, available through a website to facilitate BM risk self‐evaluation.

## AUTHOR CONTRIBUTIONS

**Ming Li:** Conceptualization (lead); methodology (lead); writing – original draft (equal). **Wenqian Yu:** Conceptualization (equal); data curation (equal); software (equal); writing – original draft (equal). **Chao Zhang:** Data curation (equal); software (equal); writing – review and editing (equal). **Huiyang Li:** Data curation (equal); writing – review and editing (equal). **Xiuchuan Li:** Validation (equal); writing – review and editing (equal). **Fengju Song:** Validation (equal); writing – review and editing (equal). **Shiyi Li:** Validation (equal). **Guoheng Jiang:** Validation (equal). **Hongyu Li:** Validation (equal). **Min Mao:** Conceptualization (equal); project administration (equal); writing – review and editing (equal). **Xin Wang:** Conceptualization (equal); project administration (equal); writing – review and editing (equal).

## FUNDING INFORMATION

This work was supported by the Natural Science Foundation of China (81903398, 81702161, 81801781, 81802508, 8191101553), the Natural Science Foundation of Tianjin Science and Technology Committee China (17JCQNJC11000), Chongqing Natural Science Foundation Program (cstc2019jcyjmsxmX0466), the Fundamental Research Funds for the Central Universities (YJ2021112), “From 0 to 1” innovation project of Sichuan University (2023SCUH0026), Sichuan Provincial Science and Technology Department central guide local science and technology development special project (2023ZYD0097), Sichuan Outstanding Youth Science Fund Project (2023NSFC1927), Sichuan Medical Association Youth Innovation Project (Q2016), 2023 Sichuan University graduate education teaching reform project (GSSCU2023043), The Foundation of Science and Technology of Sichuan Province (2021YFS0101).

## CONFLICT OF INTEREST STATEMENT

All authors have no conflict of interest to disclose.

## ETHICS STATEMENT

Ethics approval and consent to participate: Cancer is a reportable disease in every state of the USA and the data in the SEER database does not need informed patient consent.

## CONSENT

Written informed consent for publication was obtained from all participants.

## Supporting information


Appendix S1:

Appendix S2:

Appendix S3:

Appendix S4:

Appendix S5:

Appendix S6:

Appendix S7:

Appendix S8:

## Data Availability

Our data can be accessed through the following link: (https://pan.baidu.com/s/1KK5r‐aMmlRTkXDhcF‐w0IA?pwd=iceq).
