# Supplementary figures and images for: Reclassified the phenotypes of cancer types and construct a nomogram for predicting bone metastasis risk: A pan‐cancer analysis
Source: Cancer Med. 2024 Mar 1;13(3):e7014. doi: 10.1002/cam4.7014 (PMC10905679; doi:10.1002/cam4.7014)

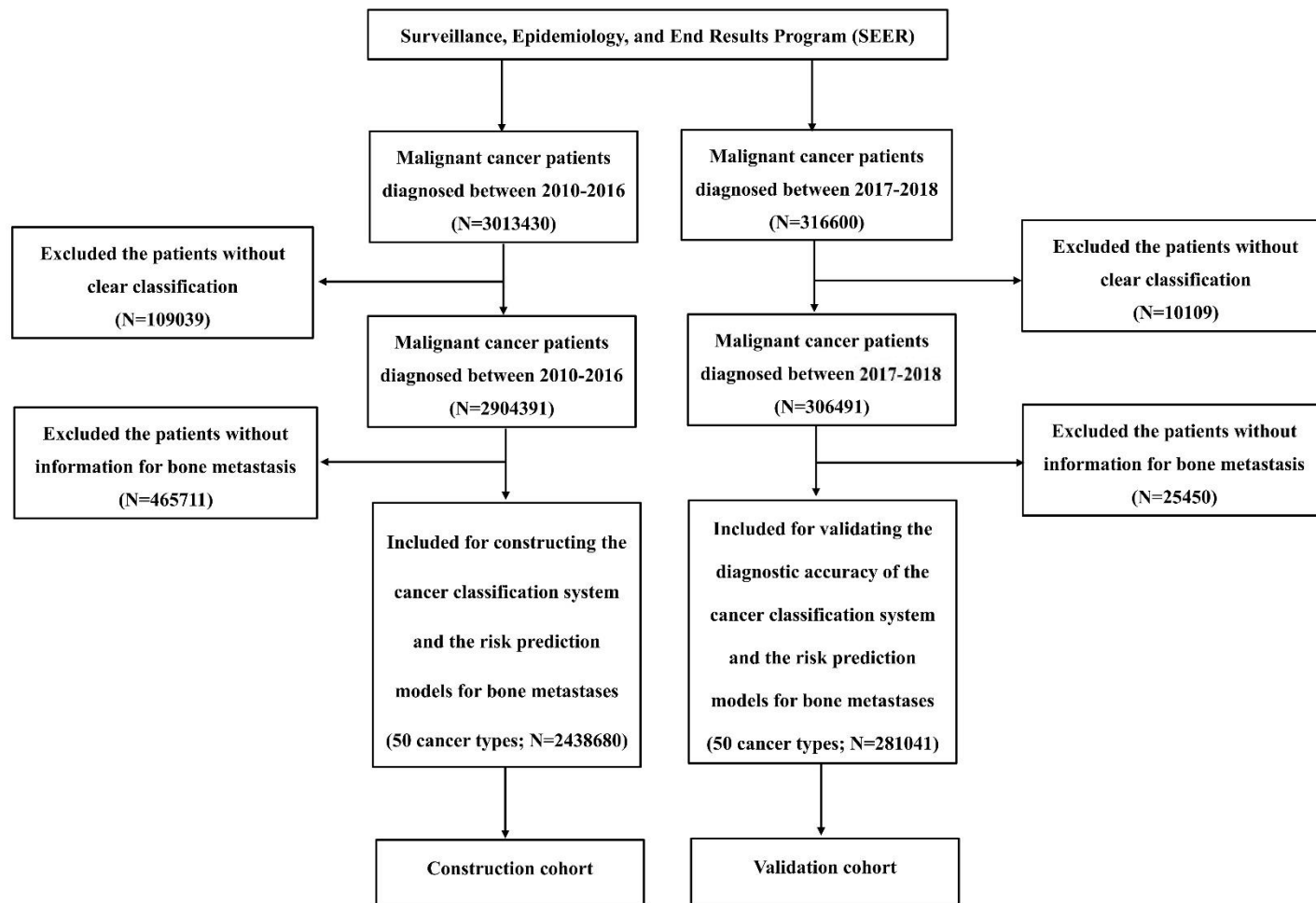

**Appendix file 1: Flow-chart for the patients selection.**

Supplement: Supplementary file 1 — Appendix S1: [file CAM4-13-e7014-s007.pdf]
