# Supplementary material for: Reclassified the phenotypes of cancer types and construct a nomogram for predicting bone metastasis risk: A pan‐cancer analysis
Source: Cancer Med. 2024 Mar 1;13(3):e7014. doi: 10.1002/cam4.7014 (PMC10905679; doi:10.1002/cam4.7014)
Supplement: Supplementary file 2 — Appendix S2: [file CAM4-13-e7014-s005.pdf]

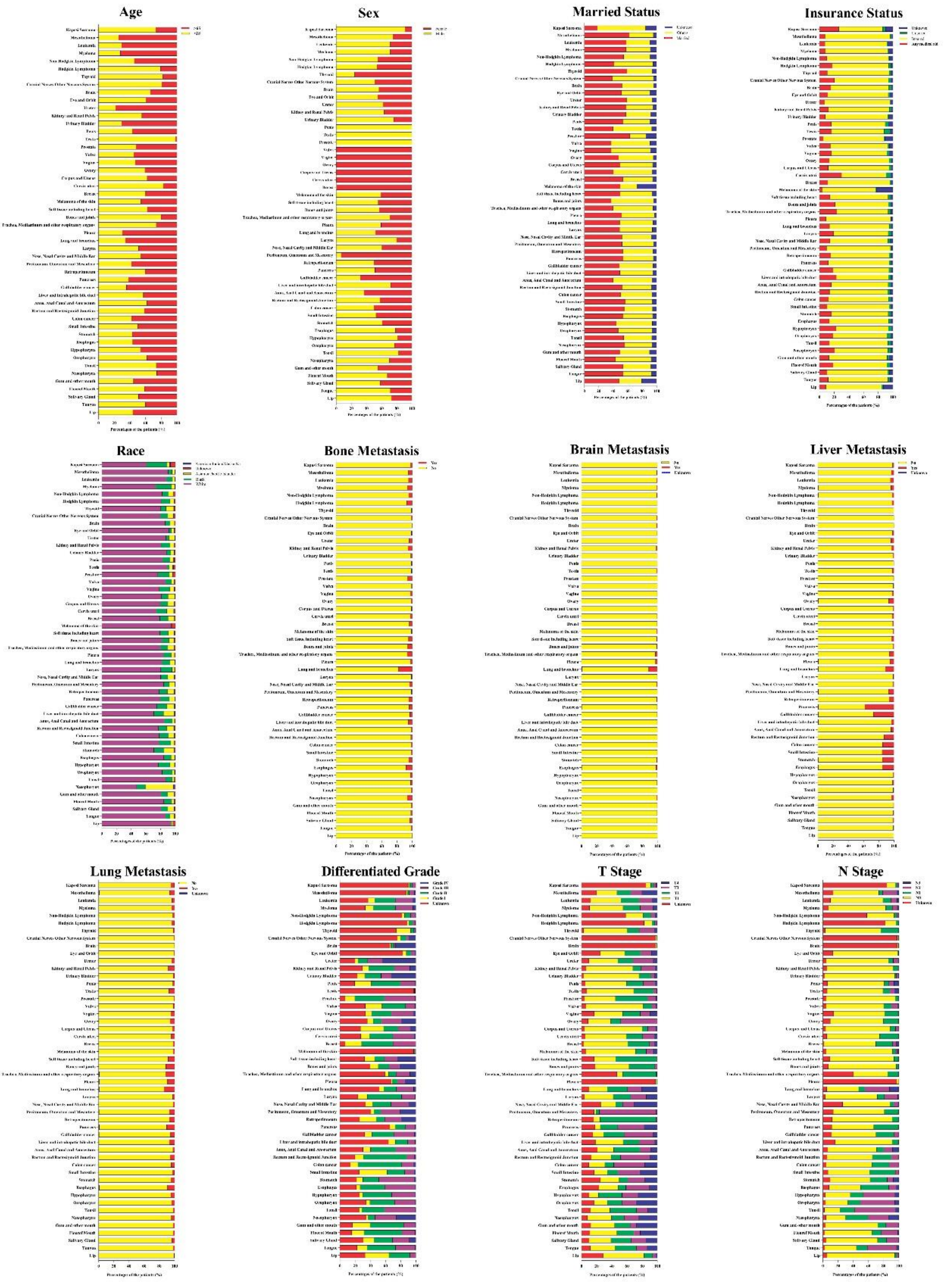

Appendix-2: Distribution of demographic and clinical characteristics for the included patients in the construction cohort. The figure describes the distributions of the demographic characteristics of age, sex, marital status, insurance status, race, and the clinical factors of brain, liver, and lung metastasis and the differentiated grade; T stage, N stage among the 50 included cancer types.
