# Supplementary material for: Reclassified the phenotypes of cancer types and construct a nomogram for predicting bone metastasis risk: A pan‐cancer analysis
Source: Cancer Med. 2024 Mar 1;13(3):e7014. doi: 10.1002/cam4.7014 (PMC10905679; doi:10.1002/cam4.7014)
Supplement: Supplementary file 3 — Appendix S3: [file CAM4-13-e7014-s006.pdf]

Appendix file 3: The differences in demographic and clinical characteristics between the construction and validation dataset.

Factors	2010-2016 N (%)	2017-2018 N (%)	Chi-square/ Z value	*P-value*
**Age (years)**			731.01	<0.001
≤65	1248635(51.2)	136328(48.5)		
>65	1190045(48.8)	144710(51.5)		
**Gender**			6.89	0.01
Male	1203836(49.4)	139467(49.6)		
Female	1234844(50.6)	141571(50.4)		
**Race**			1952.10	<0.001
White	1962889(81.6)	219206(79.3)		
Black	264289(11.0)	30189(10.9)		
Asian or pacific islander	164808(6.8)	24899(9.0)		
American Indian	14611(0.6)	2236(0.8)		
**Married status**				
None married	927767(41.6)	-		
Married	1304841(58.4)	-		
**Insurance**				
Uninsured	56887(2.5)	-		
Medical aid	277236(12.0)	-		
Insured	1983236(85.6)	-		
**Differentiated Grade**			52.59	<0.001
Well differentiated	296610(18.0)	37607(22.9)		
Moderate differentiated	702532(42.7)	71300(43.5)		
Poor differentiated	536076(32.6)	44153(26.9)		
Undifferentiated	111621(6.8)	10866(6.6)		
**T stage**			10.50	<0.001
T1	1033435(47.0)	111862(48.2)		
T2	563781(25.6)	58862(25.4)		
T3	400662(18.2)	40066(17.3)		
T4	200595(9.1)	21335(9.2)		
**Lymphatic metastasis**			14.91	<0.001
N0	1665727(73.4)	177103(74.6)		
N1	327328(14.4)	34528(14.6)		
N2	211057(9.3)	18633(7.9)		
N3	66346(2.9)	7041(3.0)		
**Bone metastasis**			114.48	<0.001

No	2314364(94.9)	265391(94.4)		
Yes	124316(5.1)	15650(5.6)		
**Brain metastasis**			1.65	0.20
No	2384501(98.1)	275069(98.1)		
Yes	47367(1.9)	5363(1.9)		
**Liver metastasis**			6.08	0.01
No	2292659(94.3)	264106(94.2)		
Yes	138930(5.7)	16344(5.8)		
**Lung metastasis**			35.33	<0.001
No	2314693(95.4)	267832(95.6)		
Yes	112119(4.6)	12244(4.4)		
