# Supplementary material for: Reclassified the phenotypes of cancer types and construct a nomogram for predicting bone metastasis risk: A pan‐cancer analysis
Source: Cancer Med. 2024 Mar 1;13(3):e7014. doi: 10.1002/cam4.7014 (PMC10905679; doi:10.1002/cam4.7014)
Supplement: Supplementary file 4 — Appendix S4: [file CAM4-13-e7014-s002.pdf]

Study name	Subgroup within study	Statistics for each study				
		Event rate	Lower limit	Upper limit	Z-Value	p-Value
Lip	Female	0.003	0.001	0.008	-10.214	0.000
Tongue	Female	0.007	0.005	0.010	-33.288	0.000
Salivary Gland	Female	0.019	0.015	0.024	-30.924	0.000
Floor of Mouth	Female	0.016	0.010	0.025	-16.865	0.000
Gum and other mouth	Female	0.011	0.008	0.015	-30.368	0.000
Nasopharynx	Female	0.044	0.034	0.058	-21.227	0.000
Tonsil	Female	0.012	0.008	0.017	-23.546	0.000
Oropharynx	Female	0.020	0.012	0.034	-13.932	0.000
Hypopharynx	Female	0.017	0.010	0.029	-13.992	0.000
Esophagus	Female	0.051	0.046	0.057	-48.010	0.000
Stomatch	Female	0.040	0.037	0.043	-80.902	0.000
Small Intestine	Female	0.013	0.011	0.016	-40.689	0.000
Colon cancer	Female	0.010	0.010	0.011	-135.962	0.000
Rectum and Rectosigmoid Junction	Female	0.013	0.011	0.014	-86.233	0.000
Anus, Anal Canal and Anorectum	Female	0.010	0.008	0.012	-39.448	0.000
Liver and intrahepatic bile duct	Female	0.036	0.034	0.040	-74.749	0.000
Gallbladder cancer	Female	0.026	0.022	0.031	-40.381	0.000
Pancreas	Female	0.032	0.030	0.034	-114.445	0.000
Retroperitoneum	Female	0.041	0.031	0.054	-21.440	0.000
Peritoneum, Omentum and Mesentery	Female	0.008	0.006	0.012	-24.174	0.000
Nose, Nasal Cavity and Middle Ear	Female	0.034	0.026	0.044	-24.693	0.000
Larynx	Female	0.008	0.005	0.011	-26.504	0.000
Lung and bronchus	Female	0.159	0.158	0.161	-242.628	0.000
Pleura	Female	0.015	0.002	0.101	-4.127	0.000
Trachea, Mediastinum and other respiratory organs	Female	0.081	0.055	0.117	-11.439	0.000
Bones and joints	Female	0.053	0.045	0.063	-31.249	0.000
Soft tissue including heart	Female	0.042	0.038	0.046	-60.534	0.000
Melanoma of the skin	Female	0.006	0.006	0.007	-97.521	0.000
Breast	Female	0.037	0.036	0.038	-404.181	0.000
Urinary Bladder	Female	0.017	0.015	0.018	-89.265	0.000
Kidney and Renal Pelvis	Female	0.048	0.046	0.050	-123.128	0.000
Ureter	Female	0.039	0.029	0.051	-21.777	0.000
Eye and Orbit	Female	0.008	0.005	0.013	-21.236	0.000
Brain	Female	0.002	0.001	0.003	-34.492	0.000
Cranial Nerves Other Nervous System	Female	0.013	0.008	0.022	-16.576	0.000
Thyroid	Female	0.006	0.005	0.006	-100.664	0.000
Hodgkin Lymphoma	Female	0.051	0.038	0.067	-19.578	0.000
Non-Hodgkin Lymphoma	Female	0.040	0.036	0.044	-62.206	0.000
Myeloma	Female	0.078	0.057	0.107	-14.192	0.000
Leukemia	Female	0.042	0.031	0.056	-19.435	0.000
Mesothelioma	Female	0.035	0.026	0.047	-22.069	0.000
Kaposi Sarcoma	Female	0.014	0.001	0.191	-2.973	0.003
Cervix uteri	Female	0.025	0.023	0.027	-85.968	0.000
Corpus and Uterus, NOS	Female	0.010	0.009	0.010	-137.802	0.000
Ovary	Female	0.011	0.010	0.013	-92.238	0.000
Vagina	Female	0.023	0.018	0.030	-27.185	0.000
Vulva	Female	0.007	0.006	0.009	-38.350	0.000
		**0.020**	**0.014**	**0.028**	**-21.998**	**0.000**
Lip	Male	0.005	0.003	0.009	-20.835	0.000
Tongue	Male	0.012	0.011	0.014	-61.594	0.000
Salivary Gland	Male	0.034	0.029	0.039	-40.873	0.000
Floor of Mouth	Male	0.016	0.011	0.022	-24.275	0.000
Gum and other mouth	Male	0.015	0.012	0.019	-36.480	0.000
Nasopharynx	Male	0.071	0.062	0.082	-34.337	0.000
Tonsil	Male	0.013	0.011	0.015	-51.875	0.000
Oropharynx	Male	0.019	0.014	0.026	-25.546	0.000
Hypopharynx	Male	0.022	0.018	0.028	-30.828	0.000
Esophagus	Male	0.087	0.083	0.091	-94.363	0.000
Stomatch	Male	0.049	0.046	0.052	-104.066	0.000
Small Intestine	Male	0.013	0.011	0.016	-43.026	0.000
Colon cancer	Male	0.015	0.015	0.016	-149.970	0.000
Rectum and Rectosigmoid Junction	Male	0.018	0.017	0.019	-110.762	0.000
Anus, Anal Canal and Anorectum	Male	0.012	0.009	0.016	-32.124	0.000
Liver and intrahepatic bile duct	Male	0.053	0.051	0.055	-127.177	0.000
Gallbladder cancer	Male	0.034	0.027	0.042	-29.109	0.000
Pancreas	Male	0.043	0.041	0.045	-123.053	0.000
Retroperitoneum	Male	0.048	0.037	0.062	-21.982	0.000
Peritoneum, Omentum and Mesentery	Male	0.037	0.019	0.072	-9.043	0.000
Nose, Nasal Cavity and Middle Ear	Male	0.031	0.025	0.039	-30.220	0.000
Larynx	Male	0.009	0.008	0.011	-56.115	0.000
Lung and bronchus	Male	0.193	0.191	0.195	-233.809	0.000
Pleura	Male	0.032	0.010	0.093	-5.835	0.000
Trachea, Mediastinum and other respiratory organs	Male	0.055	0.040	0.074	-17.530	0.000
Bones and joints	Male	0.061	0.053	0.070	-36.285	0.000
Soft tissue including heart	Male	0.051	0.047	0.055	-69.337	0.000
Melanoma of the skin	Male	0.012	0.011	0.013	-139.012	0.000
Breast	Male	0.063	0.055	0.072	-38.181	0.000
Prostate	Male	0.057	0.056	0.057	-382.040	0.000
Testis	Male	0.009	0.007	0.010	-57.648	0.000
Penis	Male	0.010	0.007	0.014	-22.549	0.000
Urinary Bladder	Male	0.017	0.016	0.018	-160.836	0.000
Kidney and Renal Pelvis	Male	0.060	0.058	0.061	-165.741	0.000
Ureter	Male	0.037	0.029	0.046	-27.587	0.000
Eye and Orbit	Male	0.009	0.006	0.013	-23.838	0.000
Brain	Male	0.002	0.001	0.003	-38.904	0.000
Cranial Nerves Other Nervous System	Male	0.014	0.008	0.022	-16.996	0.000
Thyroid	Male	0.014	0.013	0.016	-73.938	0.000
Hodgkin Lymphoma	Male	0.082	0.067	0.100	-21.930	0.000
Non-Hodgkin Lymphoma	Male	0.050	0.046	0.054	-70.742	0.000
Myeloma	Male	0.058	0.046	0.073	-22.136	0.000
Leukemia	Male	0.047	0.039	0.056	-31.337	0.000
Mesothelioma	Male	0.052	0.045	0.059	-40.294	0.000
Kaposi Sarcoma	Male	0.027	0.014	0.050	-10.652	0.000
		**0.027**	**0.020**	**0.036**	**-22.733**	**0.000**

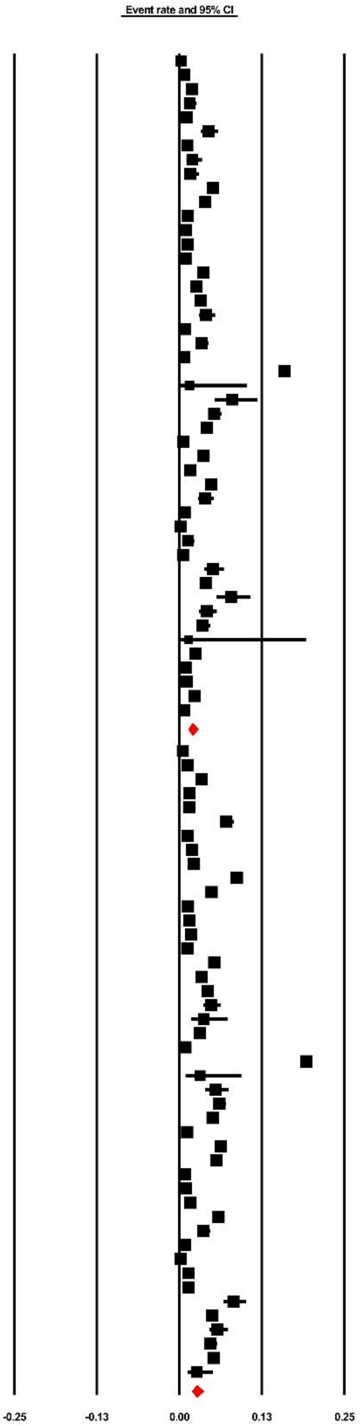

Meta Analysis

Appendix file 4: Forest plot for the pooled bone metastasis prevalence in males and females.
