# Supplementary material for: Reclassified the phenotypes of cancer types and construct a nomogram for predicting bone metastasis risk: A pan‐cancer analysis
Source: Cancer Med. 2024 Mar 1;13(3):e7014. doi: 10.1002/cam4.7014 (PMC10905679; doi:10.1002/cam4.7014)
Supplement: Supplementary file 5 — Appendix S5: [file CAM4-13-e7014-s004.pdf]

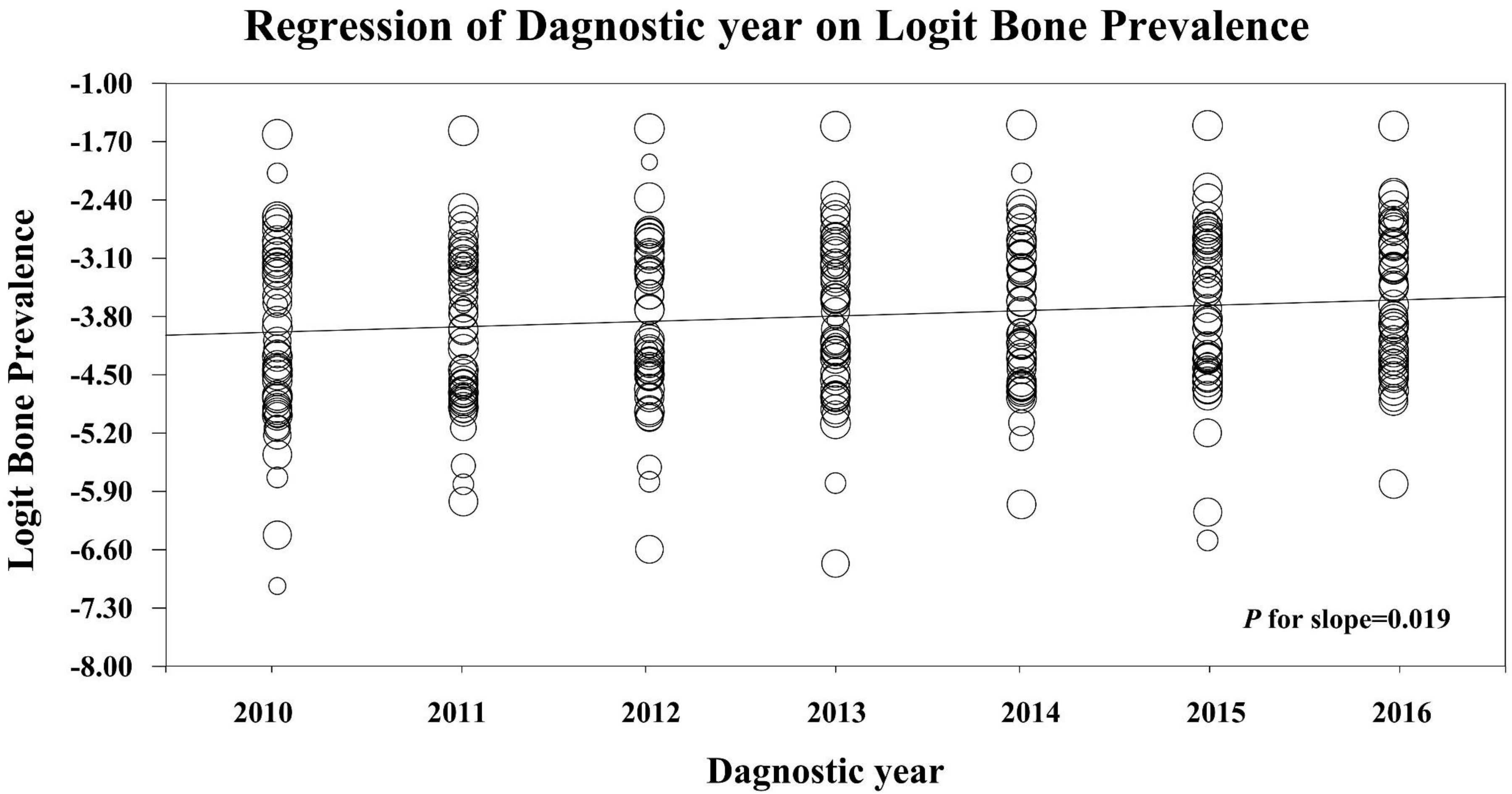

Appendix file 5: Meta-regression plot for the trend of the pooled bone metastasis prevalence changes with the diagnosed year.
