# Supplementary material for: Reclassified the phenotypes of cancer types and construct a nomogram for predicting bone metastasis risk: A pan‐cancer analysis
Source: Cancer Med. 2024 Mar 1;13(3):e7014. doi: 10.1002/cam4.7014 (PMC10905679; doi:10.1002/cam4.7014)
Supplement: Supplementary file 6 — Appendix S6: [file CAM4-13-e7014-s003.pdf]

Appendix file 6: The differences in the distribution of the associated factors for bone metastatic patients diagnosed 2010-2016.

Factors	Category A N (%)	Category B N (%)	Category C N (%)	Chi-square/ Z value	*P-value*
**Age (years)**				11479.41	<0.001
≤65	188771(44.6)	655595(50.8)[a]	424872(54.8) [ab]		
>65	234302(55.4)	634455(49.2)[a]	349889(45.2) [ab]		
**Gender**				12267.17	<0.001
Male	195259(46.2)	681193(52.8)[a]	353197(45.6)[ab]		
Female	227814(53.8)	608857(47.2)[a]	421564(54.4)[ab]		
**Race**				6918.44	<0.001
White	344242(81.8)	1015801(79.9)[a]	642437(84.3)[ab]		
Black	44302(10.5)	156068(12.3)[a]	68779(9.0)[ab]		
Asian or pacific islander	30030(7.1)	92363(7.3)[a]	46125(6.1)[ab]		
American Indian	2400(0.6)	7628(0.6)[a]	4933(0.6)[ab]		
**Married status**				11118.15	<0.001
None married	184728(46.1)	453372(38.4)[a]	310097(44.7)[ab]		
Married	216131(53.9)	728752(61.6)[a]	384274(55.3)[ab]		
**Insurance**				5339.05	<0.001
Uninsured	10530(2.5)	24724(2.0)[a]	22887(3.2)[ab]		
Medical aid	55989(13.5)	134772(11.0)[a]	92605(12.8)[ab]		
Insured	347062(83.9)	1067344(87.0)[a]	607854(84.0)[ab]		
**Differentiated Grade**				7726.01	<0.001
Well differentiated	36490(20.0)	179185(17.3)[a]	83317(18.6)[ab]		
Moderate differentiated	58434(32.0)	425480(41.1)[a]	224164(50.1)[ab]		
Poor differentiated	76864(42.1)	363961(35.2)[a]	102229(22.8)[ab]		
Undifferentiated	10676(5.9)	65866(6.4)[a]	38043(8.5)[ab]		
**T stage**				74638.36	<0.001
T1	129383(33.8)	607537(51.5)[a]	304902(46.4)[ab]		
T2	102109(26.7)	358360(30.4)[a]	108843(16.6)[ab]		
T3	79058(20.7)	152922(13.0)[a]	173702(26.5)[ab]		
T4	72066(18.8)	61437(5.2)[a]	69157(10.5)[ab]		
**Lymphatic metastasis**				181162.98	<0.001
N0	204700(51.2)	975989(80.8)[a]	503001(73.2)[ab]		
N1	47633(11.9)	175864(14.6)[a]	109209(15.9)[ab]		
N2	107708(26.9)	35895(3.0)[a]	68278(9.9)[ab]		
N3	40048(10.0)	19798(1.6)[a]	6577(1.0)[ab]		
**Brain metastasis**				133620.64	<0.001
No	382251(91.0)	1280980(99.5)[a]	769058(99.4)[ab]		
Yes	37966(9.0)	5854(0.5)[a]	4367(0.6)[ab]		
**Liver metastasis**				13436.48	<0.001
No	381416(90.8)	1228960(95.5)[a]	723388(93.6)[ab]		
Yes	38811(9.2)	57775(4.5)[a]	49844(6.4)[ab]		
**Lung metastasis**				50420.14	<0.001

No	370318(88.6)	1243993(96.8)[a]	744670(96.4)[ab]
Yes	47578(11.4)	41166(3.2)[a]	27474(3.6)[ab]

Note: a: reached significant level compared with Category A; b: reached significant level compared with Category B
