# Supplementary material for: Reclassified the phenotypes of cancer types and construct a nomogram for predicting bone metastasis risk: A pan‐cancer analysis
Source: Cancer Med. 2024 Mar 1;13(3):e7014. doi: 10.1002/cam4.7014 (PMC10905679; doi:10.1002/cam4.7014)
Supplement: Supplementary file 7 — Appendix S7: [file CAM4-13-e7014-s001.pdf]

Appendix file 7: Univariable and Multivariable Logistic regression analysis for the risk factors for bone metastasis occurrence.

Factors	Non-Bone Metastasis N (%)	Bone Metastasis N (%)	Univariable Logistic regression		Multivariable Logistic regression	
			OR (95%CI)	*P-value*	OR (95%CI)	*P-value*
**Age (years)**				<0.001		
<65	1193642(51.6)	54993(44.2)	1.00		1.00	
≥65	1120722(48.4)	69323(55.8)	1.34(1.33-1.36)		1.16(1.14-1.19)	<0.001
**Gender**				<0.001		
Male	1131729(48.9)	72107(58.0)	1.00		1.00	
Female	1182635(51.1)	52209(42.0)	0.69(0.68-0.70)		0.70(0.68-0.71)	<0.001
**Race**						
White	1864576(81.7)	98313(79.3)	1.00		1.00	
Black	248296(10.9)	15993(12.9)	1.22(1.20-1.24)	<0.001	1.06(1.03-1.10)	<0.001
Asian or pacific islander	155932(6.8)	8876(7.2)	1.08(1.06-1.10)	<0.001	0.92(0.88-0.96)	<0.001
American Indian	13841(0.6)	770(0.6)	1.06(0.98-1.14)	<0.001	0.87(0.75-1.00)	<0.001
**Married status**				<0.001		
Others	872520(41.3)	55247(46.7)	1.00		1.00	
Married	1241789(58.7)	63052(53.3)	0.80(0.79-0.81)		0.88(0.86-0.90)	<0.001
**Insurance**						
Uninsured	52449(2.3)	4438(3.7)	1.00		1.00	
Medical aid	258106(11.8)	19130(15.8)	0.88(0.85-0.91)	<0.001	0.88(0.82-0.94)	<0.001
Insured	1885347(85.9)	97889(80.5)	0.61(0.60-0.63)	<0.001	0.74(0.70-0.79)	<0.001
**Differentiated Grade**						
Well differentiated	293786(18.5)	2824(5.1)	1.00		1.00	
Moderate differentiated	688273(43.2)	14259(25.9)	2.16(2.07-2.25)	<0.001	1.46(1.39-1.53)	<0.001
Poor differentiated	502602(31.6)	33474(60.9)	6.93(6.67-7.20)	<0.001	2.81(2.67-2.94)	<0.001

Undifferentiated	107183(6.7)	4438(8.1)	4.31(4.11-4.52)	<0.001	2.76(2.60-2.94)	<0.001
**T stage**						
T1	1015780(48.3)	17655(18.5)	1.00		1.00	
T2	536989(25.5)	26792(28.1)	2.87(2.82-2.93)	<0.001	1.49(1.44-1.53)	<0.001
T3	378546(18.0)	22116(23.2)	3.36(3.29-3.43)	<0.001	1.39(1.34-1.44)	<0.001
T4	171707(8.2)	28888(30.3)	9.68(9.49-9.87)	<0.001	2.39(2.30-2.48)	<0.001
**Lymphatic metastasis**						
N0	1628718(75.3)	37009(34.8)	1.00		1.00	
N1	303170(14.0)	24158(22.7)	3.51(3.45-3.57)	<0.001	2.68(2.60-2.75)	<0.001
N2	180764(8.4)	30293(28.4)	7.38(7.26-7.49)	<0.001	2.55(2.47-2.64)	<0.001
N3	51325(2.4)	15021(14.1)	12.88(12.61-13.15)	<0.001	3.49(3.34-3.64)	<0.001
**Brain metastasis**						
No	2281034(98.7)	103467(86.4)	1.00		1.00	
Yes	31123(1.3)	16244(13.6)	11.51(11.28-11.74)	<0.001	2.84(2.71-2.97)	<0.001
**Liver metastasis**						
No	2204487(95.4)	88172(73.2)	1.00		1.00	
Yes	106578(4.6)	32352(26.8)	7.59(7.48-7.70)	<0.001	4.52(3.47-4.67)	<0.001
**Lung metastasis**						
No	2227995(96.5)	86698(72.8)	1.00		1.00	
Yes	79732(3.5)	32387(27.2)	10.44(10.29-10.59)	<0.001	4.36(4.22-4.50)	<0.001
**Group**						
Category A	271860(11.7)	58350(46.9)	1.00		1.00	
Category B	1100371(47.5)	54385(43.7)	0.23(0.23-0.24)	<0.001	0.68(0.66-0.70)	<0.001
Category C	942133(40.7)	11581(9.3)	0.06(0.05-0.07)	<0.001	0.14(0.13-0.15)	<0.001
