# Supplementary material for: Reclassified the phenotypes of cancer types and construct a nomogram for predicting bone metastasis risk: A pan‐cancer analysis
Source: Cancer Med. 2024 Mar 1;13(3):e7014. doi: 10.1002/cam4.7014 (PMC10905679; doi:10.1002/cam4.7014)
Supplement: Supplementary file 8 — Appendix S8: [file CAM4-13-e7014-s008.pdf]

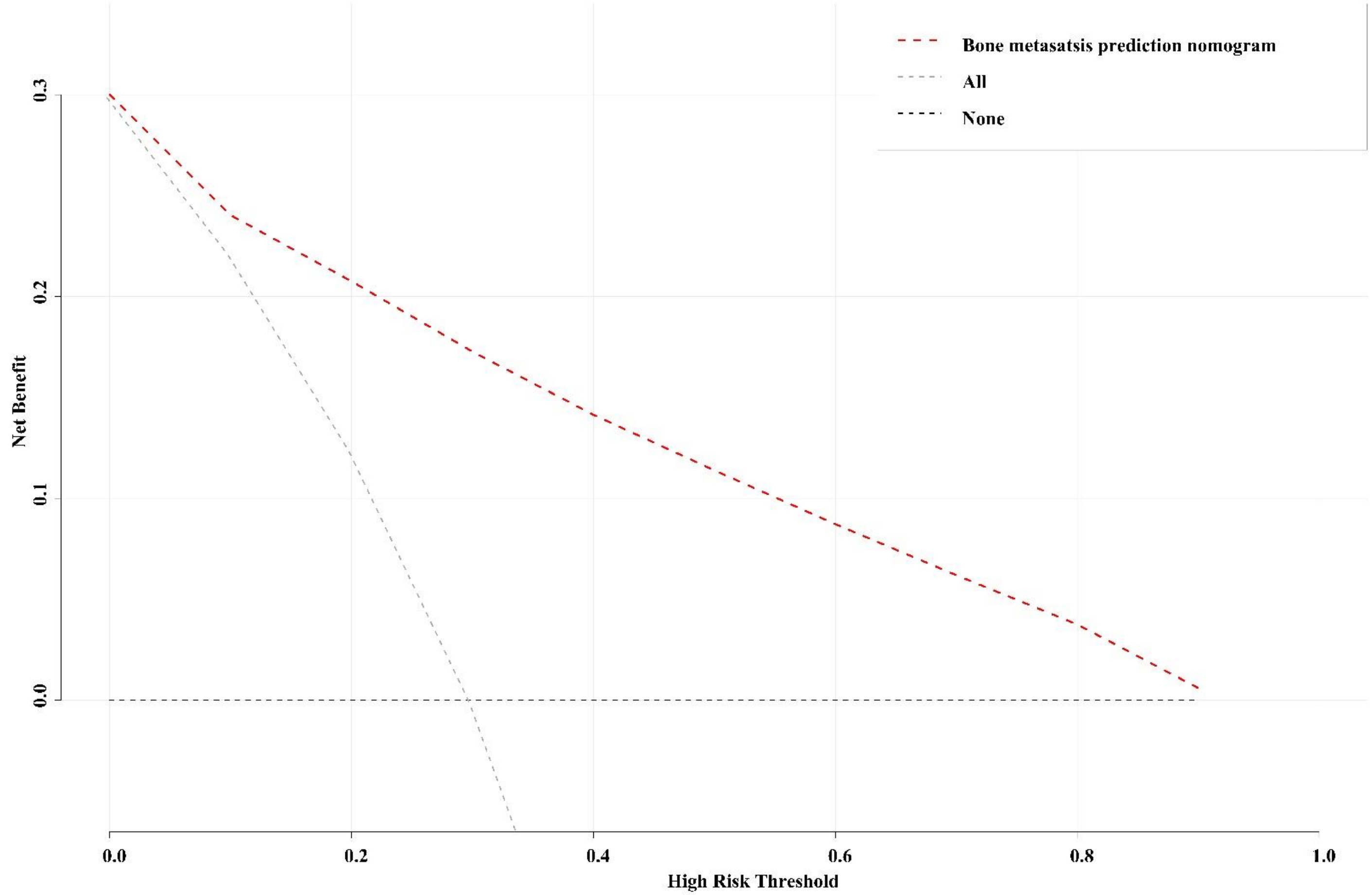

Appendix file 8: Decision curve analysis for evaluating clinical usefulness. The X-axis is the probability threshold. When a probability threshold is used as the cutoff value, the corresponding Y-axis value indicates the net benefit value, suggesting how many people per 100 people will benefit without negatively influencing other patients.
